# The Contract System in War-Time

**Published:** 1915-03-27

**Authors:** 


### THE CONTRACT SYSTEM IN WAR-TIME.

### How it is Working and the Future Outlook.

In normal times there are but few hospitals that do not buy chief supplies of food, fuel, drugs, and dressings by contract. Those not making use of this system are generally institutions with a small number of beds and a moderate resident staff. The advantages of the contract system lie chiefly in the advantageous prices brought about by competition, convenience in checking bills, and the probability that continuous custom will ensure careful attention on the part of the tradesman. These advantages are so great that it is difficult to understand how a hospital requiring a sufficient quantity of goods to make it worth while to contract can justify purchase at current rates, which in most cases would be retail prices. To buy otherwise satisfactorily would entail a knowledge of current prices constantly refreshed by a study of market reports, or the daily attendance of a qualified buyer at the chief markets. Should a hospital buy from retail dealers on the same lines as would a private household, the expenditure under some headings would be wastefully heavy. Take, for instance, milk, the cost of which is not often less than 20 per cent. of a hospital's expenditure on provisions. The general retail price is 4d. per quart, or 1s. 4d. per gallon, but the contract price for, say, 20,000 gallons and upwards annually has for the last few years ranged between 8d. and 11d. Even at the liberal price of 1s. the saving in the quantity named would be £333 6s. 8d.

The general practice in hospitals seems to have been to purchase by contract everything of which a regular supply was needed from day to day, or from week to week, and to obtain competitive tenders for goods such as linen, mackintosh sheeting, and hardware, of which a purchase was only made once in a long period. Of daily requirements greengrocery was the only item left to local retail buying.

Each contract was carefully worded, a bond for due performance was required in the case of a new or unknown contractor, goods were to be the best of their respective kinds, defective articles were to be taken back, and in the event of failure to supply goods duly ordered by the representative of a hospital, the needful quantity was to be purchased elsewhere, and the difference in price, if any, charged against the contractor. The period covered was generally six months or a year; often a year in the cases of milk or coal, where winter and summer prices are so divergent—this in order to spread expenditure over the twelve months. Commencing dates varied in different institutions. In many a hospital the same tradesman would hold a contract year after year, often being successful when his prices were not the lowest because the supply had been satisfactory, and the small difference in cost did not justify the risk of a change. These contracts were often between parties who had had business relations and known each other for many years. Probably neither side could have quoted without reference the major part of the clauses agreed to. In hardly any case (probably never except in the case of a coal contract) was war, civil commotion, strike, or other upheaval specially provided for.

Small wonder, then, when war broke out, and the first flurry and uncertainty were over, that in the majority of cases the hospitals did not insist on their full legal rights and press too heavily on those who had served them faithfully in the past. The contract being on the lines detailed above, the legal reading of the difficulty was very clear—namely, that the only cause which could excuse performance was physical impossibility to carry out the contract. Hospitals, then, were entitled to hold the contractors to the agreed terms. Happily, it was generally the case that matters were settled amicably. Many well-known firms, to their great credit, continued supply without making application to their contractees until conditions became too ruinous. Others, and these examples will also be remembered by hospital managers (but in another way), were grasping and unreasonable to a surprising degree; one firm in particular, with a large warehouse full of non-perishable goods, made short deliveries from the first day of the war, cut off the telephone, would answer neither letter nor telegram, and notified its contractees that all goods would be increased in price, whether bought under contract or in other manner. So thorough was the system immediately adopted that for sixpennyworth of hearthstone one shilling was charged on the second day of the war. Needless to say, in this case a modified position was quickly adopted, but the result will be that a lucrative connection will be lost through the unreasonable attitude first adopted.

But the contracts made before the war are running out, and the compromise prices have mostly yielded place to the higher prices of the day. As these contracts expire both hospital and tradesman are shy of entering into undertakings which involve looking forward many months; the thing is too much of a gamble, and hesitancy causes little wonder when we look at last year's prices side by side with those of to-day. Let us examine a few examples supplied by one of the larger London hospitals:

| | Contract, 1914 | Recent Purchases |
|---|---|---|
| Chilled American beef | 5½d. lb. | 8½d. lb. |
| New Zealand mutton (shoulders) | 5¼d. lb. | 7d. lb. |
| Milk (12 months) | 10d. gallon | 1s. gallon |
| Bread | 10s. 5d. cwt. | 16s. 4d. cwt. |
| House coal | 17s. 10d. ton | about 30s. ton |
| Bacon | 9d. lb. | 10½d. lb. |
| Loaf sugar | 17s. 6d. cwt. | 30s. 6d. cwt. |
| Acid. acetyl. salicyl. | 1s. 9½d. lb. | 7s. 6d. lb. |
| Bismuth carb. | 8s. 9½d. lb. | 11s. 5d. lb. |
| Phenacetin | 2s. 10d. lb. | 7s. lb. |
| Sod. bromid | 1s. 10d. lb. | 3s. lb. |
| B.P. carbolic ice crystals | 3⅞d. lb. | 1s. 4½d. lb. |

And for goods not bought under contract—linen, mackintosh, firewood, bedsteads, greengrocery, for almost everything one can think of—an enhanced price has to be paid. And when we pay war prices we sometimes wonder if the war is responsible for everything or, rather, if the burden is being fairly divided between the seller and the buyer. Some of us have an unhappy memory of the way in which the whole of the employer's portion of payments under the National Insurance Act was unblushingly lifted in many trades on to the price of goods. No wonder, then, that the suspicion may arise that direct war taxation is also to an undue extent placed upon the shoulders of the consumer of foodstuffs and drugs.

To return to practical considerations, it is necessary to consider what is best to be done in the matter of contracts. Last year, six months seemed a short time for a bond of this character. Now six months may bring about enormous fluctuations in prices. Relief of the heavy pressure on our railway systems will cause a fall of several shillings per ton in coal; the opening of the Dardanelles will bring large quantities of grain to the English market; and speeding up of the manufacture of equivalents to German drugs will lessen the expenditure of our dispensaries. Forward purchases, except in rare instances, appear to be inadvisable, and it is only in the cases of some goods (drugs, etc.) where markets are limited that a hand-to-mouth policy does not seem the best. This condition, of course, greatly increases the work of those responsible for the purchase of supplies, frequent reference to market reports and comparison of rates with other institutions are more than ever necessary. Here and there, with such articles as bread and meat, tenders for monthly periods may with a certain amount of safety be invited and considered. It is probable that the acceptance of such contracts may be profitable, for the tradesman, with an eye to future dealings, will quote reasonable prices. It is, however, except in very rare instances, not the time for contracts for longer periods.

And while this state of things prevails many of the hospitals have more patients to feed and more beds to furnish with linen. When at the end of the year we look at the recorded statistics we shall find that the average daily occupation of beds has been higher than at any time within the memory of hospital managers. This will be so because our voluntary system is bearing the double strain of doing its utmost for the civil population, and at the same time helping our brave Army by looking after every wounded soldier it is possible to make room for. Hospital managers are faced by a grave financial problem, but they are optimists for a good reason—their experience tells them that the generosity of the public never fails them in an emergency, and they have never been in a better position to ask, or had a better case to present, than at present.

Money will come without fail, but however full or empty our treasury may be, the hospitals must act up to their best traditions in carefully expending money placed at their disposal.

